# Prebiotic metabolic networks?

**DOI:** 10.1002/msb.20145351

**Published:** 2014-04-25

**Authors:** Pier Luigi Luisi

**Affiliations:** ^1^ Department of BiologyUniversity of Roma TreRomeItaly

## Abstract

A prebiotic origin of metabolism has been proposed as one of several scenarios for the origin of life. In their recent work, Ralser and colleagues (Keller *et al*, 2014) observe an enzyme‐free, metabolism‐like reaction network under conditions reproducing a possible prebiotic environment.

The question of the origin of life is currently dominated by the quest of mechanisms explaining the emergence of self‐replicating and self‐organizing systems. A majority of contemporary studies deal therefore with sophisticated processes of RNA formation and self‐replication, while the field has turned away from exploring the equally crucial question of the prebiotic origins of metabolism, as initially advocated by Morowitz *et al* (1991) or Wächtershäuser (1990). Which came first? Metabolism or enzymes (Fig 1)? This question has been sometimes heavily debated (Orgel, 2000), backdropped with the vexing question of the origin of RNA and its sugar–phosphate backbone. Of course, nobody knows who is right. The recent study by Ralser and collaborators reporting the observation of abiotic metabolic‐like reactions provides thus most welcome new empirical data that will stimulate a thought‐provoking discussion on the very first steps of metabolism (Keller *et al*, 2014).

**Figure 1:**
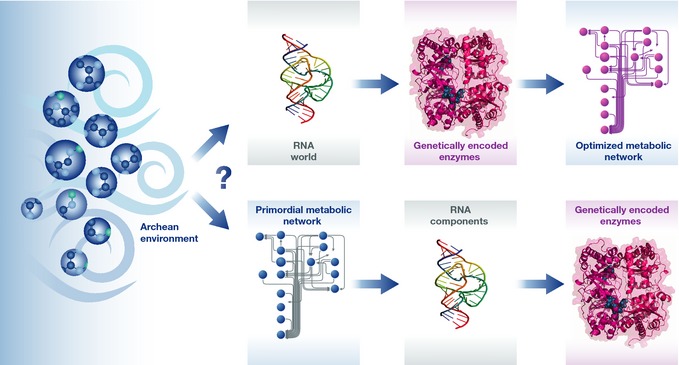
The unknown origin of metabolism Are metabolic pathways the result of the evolutionary selection of genetically encoded catalysts (enzymes)—and thus appeared as consequence of genetics which became possible in the RNA world (top row)—or does metabolism date back to chemical reaction sequences based on a prebiotic chemistry, which then facilitated the emergence of RNA components and, therefore, of genetics (bottom row)?

The core structure of the metabolic network is very similar across all organisms, which suggests an early origin during evolution (Jeong *et al*, 2000). Centrally located within this network are the sugar phosphate reactions of glycolysis and the pentose phosphate pathway. Together with the overlapping reactions of the Entner–Doudoroff pathway and of the Calvin cycle, they provide the precursor metabolites required for the synthesis of RNA, DNA, lipids, energy and redox coenzymes and amino acids—key molecules required for life. Investigating the hypothesis that metabolic‐like reactions could have already emerged in a prebiotic world, Ralser and colleagues systematically explored whether non‐enzymatic interconversion of sugar phosphate metabolites is experimentally detectable under conditions resembling that of the (average) Archean ocean. Starting from metabolites of glycolysis and the pentose phosphate pathway, such as glucose 6‐phosphate or fructose 6‐phosphate, a total to 29 metabolism‐like reactions were observed. In these experiments, under anoxic conditions and after 5 h at 70°C, more than half of the carbon was recovered as glycolytic and pentose phosphate intermediates, showing that these abiotic reactions represent a significant proportion of the chemical interconversion activity occurring in this ‘primordial soup’. Furthermore, investigating the impact of the putative ionic composition of the Archean ocean, as inferred from geological records, Ralser and colleagues observed that ferrous iron [Fe(II)] stimulates the speed and specificity of what appears a network of reactions that remarkably overlaps central carbon metabolism of living organisms.

The study by Keller *et al* shows that physico‐chemical constraints might be at the origin of the structure of biological metabolic networks. In addition, simple metallic molecules that were likely found in the prebiotic environment can act as catalysts for reactions that depend on sophisticated enzymes in modern cells. Of course, the study does not start from ground zero. To the question ‘Where did these sugars come from?’, we can only speculate based on the prior literature, which leaves the issue essentially open. Nonetheless, the mere fact that these reactions occur under plausible prebiotic conditions obliges us to really think in true prebiotic terms.

The notion of the prebiotic Archean *ocean* is intriguing, particularly when considering the problem of concentration of the solutes. It is indeed unlikely that life started in such conditions of extreme dilution of a few molecules in the prebiotic ocean, and some form of compartmentalization might be considered to explain how the necessary local metabolite concentration was achieved. Another important question, which is otherwise rarely considered in the literature, relates to the role of self‐replication mechanisms as opposed to reactions under thermodynamic control. Indeed, there is no need, in principle, of self‐replication, when a given product is continuously re‐made because it is the most stable reaction product—this is the case of all reactions under thermodynamic control. Related to this issue is the fact that thermodynamically less favored molecules can in fact accumulate as well if products are separated from the catalyst, for example, by inclusion into protocell vesicles. Thus, it cannot be excluded that a primordial ‘reversed’ glycolysis (that is, a primordial ‘gluconeogenesis’) could have contributed to carbon fixation or facilitated the formation of the complex sugar phosphates required for the assembly of RNA and other biological molecules. Whether this was the case is not known at present, but these hypotheses are just a few of the many stimulating research avenues that will be spurred by this technically and conceptually stimulating work.

## Conflict of interest

The author declares that he has no conflict of interest.
